# The effects of dose, valency, and affinity on TfR-mediated brain delivery in vivo

**DOI:** 10.1186/s12987-025-00643-y

**Published:** 2025-04-08

**Authors:** Gillian Bonvicini, Sunitha Singh, Lisa Sandersjöö, Dag Sehlin, Stina Syvänen, Ken G. Andersson

**Affiliations:** 1 BioArctic AB, Warfvinges Väg 35, 112 51 Stockholm, Sweden; 2 Department of Public Health and Caring Sciences/Geriatrics, Rudbeck Laboratory, Uppsala University, Dag Hammarskjölds Väg 20, 751 85 Uppsala, Sweden

**Keywords:** Transferrin receptor-mediated transcytosis, Antibody, Brain delivery, Valency, Apparent affinity, Dose

## Abstract

**Background:**

Monovalent binding to the transferrin receptor (TfR) is considered the most efficient mode for high delivery of protein constructs across the blood–brain barrier via TfR-mediated transcytosis at therapeutic doses. However, growing evidence suggests this is not the case at lower, diagnostic doses. There is also a lack of data on how valency and affinity to TfR affect brain uptake independently since previous studies have not compared monovalent and bivalent antibodies with similar affinities regardless of valency (i.e. apparent affinity). Therefore, the aim was to evaluate the independent effects of valency and affinity on TfR-mediated brain delivery at different doses.

**Methods:**

Affinity variants of antibody 8D3 were produced by introducing alanine point mutations into the complementarity-determining regions. Eleven Fab fragments and 29 IgGs were affinity screened against mouse TfR (mTfR). Six of each were chosen for production with a knob-into-hole design to have monovalent and bivalent TfR binders in full-length antibody format. The apparent affinity of these 12 antibodies were tested in an Sp2/0-Ag14 cell assay. The 10 nM apparent affinity set and the bivalent wild-type antibody were radiolabelled and injected into wild-type mice at a low (0.22 ± 0.03 mg/kg) or high (7.5 ± 0.43 mg/kg) dose. The biodistribution was measured in brain, blood and peripheral organs 4 h post-injection.

**Results:**

Two sets of monovalent and bivalent 8D3 formats with similar mTfR apparent affinities were identified. Brain and tissue uptake was higher at the low dose than the high dose for all antibodies. At the low dose, the higher apparent affinity, bivalent antibody had higher brain uptake than either of the two lower apparent affinity antibodies. At the high dose, the monovalent antibody had higher brain uptake than the two bivalent antibodies. The peripheral distribution of the three antibodies were similar to the brain distribution at both doses.

**Conclusions:**

Valency and apparent affinity affect brain uptake in a dose-dependent manner such that: brain uptake was affected more by apparent affinity at the low dose and by valency at the high dose. Thus, when designing constructs for TfR-mediated brain delivery, the application, and consequently the dose, are critical to consider.

**Supplementary Information:**

The online version contains supplementary material available at 10.1186/s12987-025-00643-y.

## Background

The blood–brain barrier (BBB) is a physiological barrier that protects the brain from peripheral substances. However, it poses significant challenges in the development of therapeutics for central nervous system (CNS) diseases since most therapies require access to the CNS to be effective [1]. Technological advancements in therapies for peripheral disorders are typically slower to gain traction for CNS diseases because of the additional hurdle of traversing the BBB. This issue has been especially true for large molecular biologics for therapies and diagnostics, including monoclonal antibodies, which do not passively cross the BBB well. Less than 0.05% of an injected dose of an antibody typically enters the brain [2–5].

Strategies to enhance brain uptake of biologics have been explored over the past decades. There are receptors present on BBB endothelial cells whose endogenous function is to carry large molecules into the brain to maintain homeostasis of essential molecules and nutrients. Several research groups have developed molecular Trojan horse strategies where a biologic drug is conjugated with an antibody that binds to these receptors [6]. Conventionally, a bispecific protein construct is administered into the blood, where it binds to the receptor on the BBB. This binding enables the transport of the construct into the brain, allowing it to be available for the CNS disease-specific target.

The transferrin receptor (TfR) is primarily responsible for maintaining iron homeostasis in the brain and has received substantial attention as an efficient shuttle for this strategy over the past two decades [7, 8]. Several factors have proven to be important for optimizing TfR-mediated transcytosis of biologics, including: the affinity towards TfR [9–16], the pH-dependency of the affinity to TfR [17, 18], the valency of the interaction with TfR [3, 6, 19–22], and the size of the bispecific protein [16]. A consensus in the field is that the antibody should bind to TfR monovalently and with an intermediate affinity for efficient transcytosis of a therapeutically dosed antibody [9, 14, 19, 20]. Antibodies with high TfR affinity have been shown to become trapped in the lysosomes while antibodies with too low affinity do not bind to TfR strongly enough to undergo efficient transcytosis [9, 11, 12, 14]. Furthermore, pH-dependent affinity, where the construct binds stronger to TfR at pH 7.4 and weaker at pH 5.5, could enhanced brain uptake due to a rapid release in the pH 5.5 endosomes, facilitating availability for release into the parenchyma [18]. Bivalent binders also have a higher likelihood of being trafficked into lysosomes than monovalent binders. It has been suggested that bivalent binders can simultaneously bind two different TfR molecules on the surface, causing receptor clustering and subsequent receptor degradation in the lysosome [19, 20]. However, previous studies on the effects of valency have utilized the same anti-TfR antibody in both bivalent and monovalent formats. This innately introduces a change in apparent affinity since most antibodies in their full, bivalent format will bind stronger due to avidity. Size has also been suggested to affect brain uptake by influencing blood pharmacokinetics and tissue elimination rates [16]. Despite these limitations, the different valency formats compared in literature often involve TfR-binding moieties attached to a therapeutic antibody or enzyme resulting in different sizes and apparent affinities between the valency formats [6, 16, 19, 20, 22].

In this study, we matched monovalent and bivalent TfR-binders of equivalent size and apparent affinity to investigate the effect of valency on transcytosis without underlying apparent affinity or size differences. Previously described alanine point mutations in the complementarity-determining regions of the anti-mouse TfR1 (mTfR) antibody, 8D3, were used to produce 12 Fab fragments and 29 IgGs with varying affinities [12, 15]. We successfully identified two sets of monovalent and bivalent 8D3 antibodies with matching apparent affinity on cells. An in vivo test with the 10 nM apparent affinity set and the bivalent wild-type (WT) 8D3 indicated that, at a similar size, brain uptake via TfR-mediated transcytosis is more affected by apparent affinity at lower doses and by valency at higher doses.

## Methods

### Recombinant antibody production

#### Design

IgG and Fab fragment affinity variants of 8D3 were produced by introducing point mutations in the complementarity-determining regions previously identified by Do and colleagues and Webster and colleagues (Table S1, S2) [12, 15, 23]. Fab fragments were designed with a 10xHis tag on the C-terminus of the heavy chain.

Monovalent and bivalent affinity variants of interest for further analysis were re-formatted into full-length knob-into-hole (KiH) IgG antibodies with S354C/T366W mutations on the knob antibody Fc and Y349C/T366S/L368A/Y407V mutations on the hole antibody Fc [24]. All full-length KiH antibodies had LALA-PG mutations (L234A/L235A/P329G) to reduce effector function [25]. Monovalent variants of interest were produced as full-length KiH IgG antibodies where an 8D3 affinity variant-derived knob antibody was paired with a Bapineuzumab (Bapi)-derived hole antibody (Table 1). Bapi binds to human amyloid-β. Since human amyloid-β is absent in WT mice, the Bapi-derived hole antibody has no target. Therefore, the monovalent Bapi-8D3 antibodies are the same size as the bivalent 8D3 antibodies allowing for the comparison of monovalent and bivalent binding to TfR without the influence of antibody size or other target binding.

**Table 1:** Knob and hole pairings and 8D3 point mutations in the bivalent and monovalent KiH antibodies

	Knob	Hole	8D3 point mutations
Bivalent 8D3 variants			
8D3_WT_	8D3_WT_	8D3_WT_	none
8D3_Y52A/Y92A_	8D3_Y52A/Y92A_	8D3_Y52A/Y92A_	Y52A/Y92A
8D3_D54A/Q90A_	8D3_D54A/Q90A_	8D3_D54A/Q90A_	D54A/Q90A
8D3_D54A/Y92A_	8D3_D54A/Y92A_	8D3_D54A/Y92A_	D54A/Y92A
8D3_Y32A/Q90A_	8D3_Y32A/Q90A_	8D3_Y32A/Q90A_	Y32A/Q90A
8D3_Y103A/Y92A_	8D3_Y103A/Y92A_	8D3_Y103A/Y92A_	Y103A/Y92A
Monovalent 8D3 variants			
Bapi-8D3_WT_	8D3_WT_	Bapi	none
Bapi-8D3_Y92A_	8D3_Y92A_	Bapi	Y92A
Bapi-8D3_Y32A_	8D3_Y32A_	Bapi	Y32A
Bapi-8D3_Y52A_	8D3_Y52A_	Bapi	Y52A
Bapi-8D3_Y52A/Y92A_	8D3_Y52A/Y92A_	Bapi	Y52A/Y92A
Bapi- 8D3_Y103A/T94A_	8D3_Y103A/T94A_	Bapi	Y103A/T94A

All constant regions and the Bapi variable regions were from human and the 8D3 variable regions were from rat. Genes were synthesized and subcloned into pcDNA3.4 plasmids by GenScript Biotech (Leiden, Netherlands) or GeneArt (ThermoFisher Scientific).

#### Production

All antibodies and Fab fragments were produced with the ExpiCHO Expression System following the manufacturer’s instructions (ThermoFisher Scientific, Stockholm, Sweden). For the bispecific antibodies, the 8D3 knob and Bapi hole antibodies were produced separately and assembled with a disulphide exchange approach. Briefly, ExpiCHO-S cells (ThermoFisher Scientific) were cultured in ExpiCHO Expression medium (ThermoFisher Scientific) at 37 °C, 120 RPM orbital shaking with 80% humidity and 8% CO_2_. ExpiCHO-S cells (6 million viable cells/mL) were transfected with plasmids (1 µg/mL of transfection volume) mixed with ExpiFectamine CHO Reagent (ThermoFisher Scientific) and OptiPRO SFM (ThermoFisher Scientific). Plasmid ratios were 1:1 heavy chain to light chain for the Fab fragments and standard IgGs, 1:1:2 8D3 knob heavy chain to 8D3 hole heavy chain to light chain for the bivalent KiH antibodies, 1:1 8D3-derived knob or Bapi-derived hole heavy chain to light chain for the knob or hole antibodies respectively. One day post-transfection, ExpiFectamine CHO Enhancer and ExpiCHO Feed were added to the flask. Cell supernatant was harvested for purification 8 d post-transfection.

#### Purification

The 12 Fab fragments were purified with HisPur™ Ni–NTA Magnetic Beads following the manufacturer’s instructions (ThermoFisher Scientific). Briefly, beads (80 µL) were washed twice with equilibration buffer (20 mM Tris, 500 mM NaCl, 0.05% Tween, pH 8). An equal volume of equilibration buffer was added to cell supernatants and the mixture was added to the tube with the beads. Samples were mixed with end-over-end rotation for 1 h at room temperature. Supernatants were discarded and the beads were washed twice with wash buffer (20 mM Tris, 500 mM NaCl, 10 mM imidazole, 0.05% Tween, pH 8). Fab fragments were eluted with a 15 min incubation in elution buffer (20 mM Tris, 500 mM NaCl, 500 mM imidazole, pH 8) on a rotating platform. Supernatants were collected and buffer exchanged to phosphate buffered saline (PBS) with Zeba spin desalting columns 40 K MWCO (ThermoFisher Scientific).

The 29 bivalent antibodies for the initial screen were purified on a KingFisher Apex (ThermoFisher Scientific) using PureProteom™ Protein G Magnetic Beads (Merck Millipore KGaA, Darmstadt, Germany). Protein G beads (100 µL) were mixed with PBS (500 µL) in a 96 well plate. Supernatants (1 mL) were added to another 96 well plate. Three wash plates were prepared with PBS (1 mL/well) and an elution plate was prepared with 0.7% acetic acid (130 µL). Plates were loaded in the KingFisher Apex. The beads were collected and released in the supernatant plate and mixed for 30 s. The beads were moved to the first wash plate and mixed for 1 min. This was repeated two more times in the other two wash plates. The beads were moved to elution plate for 2 min of mixing. Finally, the beads were moved back to the bead plate. The eluted antibodies in the elution plate were buffer exchanged to PBS with Zeba spin desalting columns 7 K MWCO (ThermoFisher Scientific).

Bivalent KiH 8D3 antibodies, 8D3 knob antibodies, and Bapi hole antibodies were purified on an ÄKTA Avant system (Cytiva, Uppsala, Sweden). Cell supernatants were applied to a HiTrap MabSelect SuRe (Cytiva) and antibodies were eluted with a linear gradient of 0.7% acetic acid. Following elution, knob and hole antibodies were stored at 4 °C until assembly while monospecific 8D3 antibodies underwent buffer exchange to PBS with a HiPrep 26/10 Desalting column (Cytiva).

#### Full-length bispecific antibody assembly

KiH bispecific antibody assembly was performed as previously described [26]. Briefly, the pH of knob and hole antibodies (13 µM) was adjusted to 8–8.5 with 120 mM L-arginine before a 1 h incubation at 35 °C. Equal parts of knob and hole half-antibodies and L-glutathione reduced (200 molar excess) were mixed and incubated for 6 h at 35 °C with end-over-end mixing. The reaction was stopped with a buffer exchange to PBS over a PD-10 desalting column (Cytiva). Bispecific antibodies were purified with an ÄKTA Purifier system (Cytiva). Samples were buffer exchanged to 20 mM TrisHCl, pH 8 with a HiPrep 26/10 desalting column in preparation for ion exchange chromatography over a HiTrap Q HP ion exchange column (Cytiva) with the elution buffer (20 mM TrisHCl and 1 M NaCl, pH 8) injected from 0–50% over 5 column volumes. Size exclusion chromatography with a HiLoad 26/600 Superdex 200 pg column (Cytiva) was performed to further purify and buffer exchange the bispecific antibodies to PBS.

#### Quality control

Protein purity was assessed with both reducing and non-reducing conditions in SDS-PAGE gels. Briefly, 1 μg of antibody was mixed with Bolt LDS Sample Buffer (ThermoFisher Scientific) (non-reducing) or with Bolt LDS Sample Buffer and NuPAGE Sample Reducing Agent (ThermoFisher Scientific) (reducing). Samples were heated for 10 min at 90 °C prior to loading on a Bolt 4–12% Bis–Tris Protein Gel (ThermoFisher Scientific). The gel was run at 200 V for 35 min with Bolt MES SDS Running Buffer (ThermoFisher Scientific), fixed in 50% methanol and 7% acetic acid and stained with InstantBlue Coomassie Protein Stain (Abcam, Cambridge, UK).

### Biacore affinity analysis

**Fig. 1:**
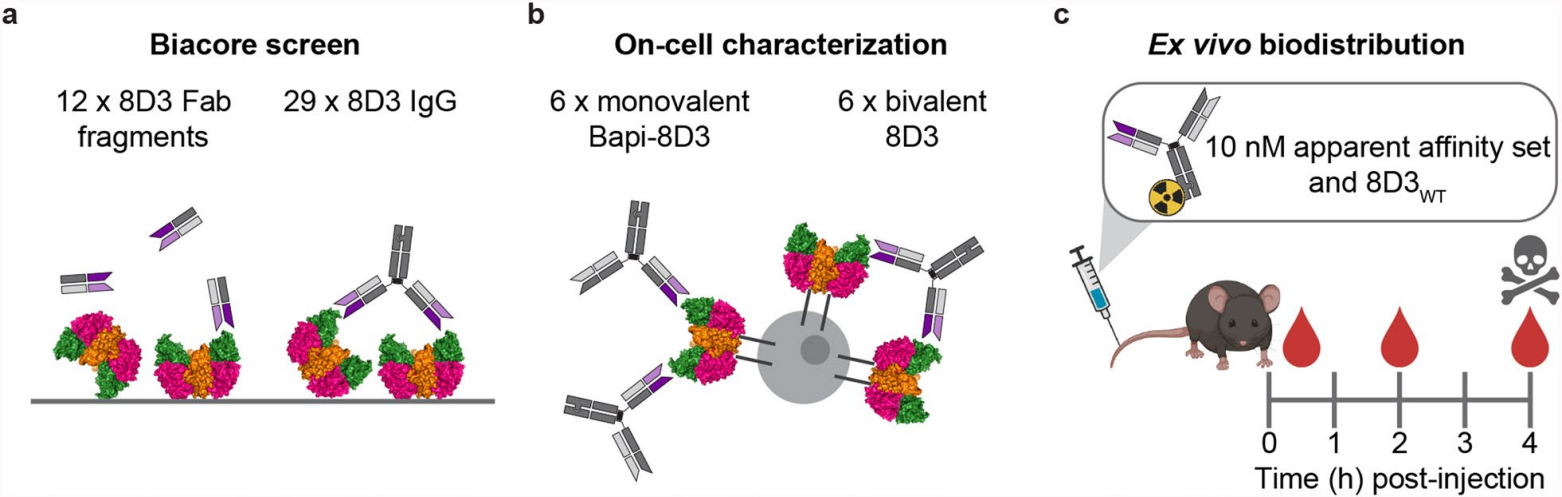
Schematic illustration of methods. **a** Affinity to mTfR of 8D3 alanine point mutation variants (12 monovalent Fab fragments and 29 bivalent IgGs) were screened in Biacore. **b** Six monovalent and six bivalent variants were selected from the initial screen and were redesigned as full-length, symmetrical KIH antibodies. The affinities of the reformatted variants to mTfR were further characterized in a cell assay. **c** A monovalent Bapi-8D3 and bivalent 8D3 with an apparent affinity of 10 nM (10 nM apparent affinity set) and the bivalent wild-type 8D3 (8D3_WT_) were radiolabelled and injected into WT mice for ex vivo biodistribution analysis

An initial affinity screen of the 12 Fab fragments and 29 IgGs was performed with a Biacore 8 K (Cytiva) as previously described (Fig. 1a) [27]. Briely, a CM5 chip (Cytiva) was immobilized with mTfR ectodomain (C89-F763 with a polyhistidine tag and TEV cleavage site at the N-terminus produced in house; 3 µg/mL in 10 mM sodium acetate, pH 5.5) on the surface of flow cell 2. Analytes were injected on both flow cells making flow cell 1 the reference cell. Data was reference and blank subtracted prior to analysis with Biacore Insight Evaluation (Cytiva, version 3.0.12.15655).

Affinities for the Fab fragments were measured with a 1:1 binding model. Fab fragments were applied in four consecutive association steps of 120 s (12.5–100 nM for 8D3_WT_ Fab, Fab 1–2, Fab 9–11; 62.5–500 nM for Fab 3–8) followed by a 1000 s dissociation step. The flow rate was 30 µL/min and surfaces were regenerated with 4 M NaCl.

Affinities for the bivalent IgGs were initially tested with the same 1:1 binding model setup as the Fab fragments but not all IgGs fit well with a 1:1 fit model. Affinities were therefore determined with steady state affinity fit model since we were mainly interested in the apparent affinity for matching purposes. For this analysis, IgGs were applied in five consecutive association steps of 600 s (3.1–250 nM for IgG 9; 5.6–450 nM for IgG 10 and 16; and 6.2–500 nM for the remaining IgGs), followed by a 30 s dissociation step. The flow rate was 15 µL/min and surfaces were regenerated with 4 M NaCl.

### On-cell mTfR affinity assay

Apparent affinity to mTfR endogenously expressed on Sp2/0-Ag14 cells (ATCC, Manassas, USA) was measured for the candidate bivalent and monovalent full-length 8D3 variants as described previously (Fig. 1b) [27]. Briefly, cells (0.3 million viable cell/well) were blocked with Fc receptor blocker (NB309, Innovex Biosciences Inc., Richmond, USA) for 20 min. Cells were incubated with antibodies for 30 min at 4 °C. Antibodies were serially diluted in concentration ranges optimized so each antibody obtained a plateau at the higher concentrations (0.002–100 nM for 8D3_WT_; 0.003–200 nM for 8D3_Y32A/Q90A_; 0.007–400 nM for Bapi-8D3_WT_; 0.008–500 nM for 8D3_D54A/Y92A_; 0.01–600 nM for Bapi-8D3_Y92A_ and Bapi-8D3_Y32A_; 0.10–6000 nM for the remaining antibodies). Cells were washed with PBS and then incubated with Alexa 488 anti-human IgG1 Fc secondary antibody (1:200 in PBS, H10120, ThermoFisher Scientific) for 30 min at 4 °C. After incubation with the secondary antibody, cells were washed and resuspended in PBS. Fluorescent signal was acquired in BD FACSLyric (BD Biosciences, San Jose, CA) and data were analysed using flowJo, version 10.8.1 (BD Biosciences). Mean fluorescent intensity (MFI) was background corrected by subtracting the MFI from control sample with only secondary. The background corrected MFI was plotted against the antibody concentration for two replicates.

### Radiochemistry

Antibodies were radioiodinated using the direct iodination Chloramine-T method [28]. Briefly, 60 ± 41 µg of antibody was mixed with 6.1 ± 1.6 MBq of iodine-125 (^125^I) stock solution (PerkinElmer Inc., Waltham, USA) and 4.5%w/v Chloramine-T (Sigma Aldrich) in a reaction volume of 55 or 110 µl. The mixture incubated for 90 s at room temperature. The reaction was stopped with the addition of 8.3%w/v sodium metabisulfite (Sigma Aldrich) so that the final reaction volume was 60 or 120 µL respectively. Radiolabelled antibodies were desalted with Zeba spin desalting columns 7 K MWCO (ThermoFisher Scientific) to remove any free iodine. The radiochemical reaction yields for each antibody are listed in Table 2. Indirect ELISAs were performed as quality control to ensure that the radiolabelled antibodies had retained their binding affinity to mTfR.

**Table 2:** Radiochemical reaction yield (%) and molar activity (MBq/nmol) for both doses for each antibody

Antibody	Reaction yield (%)	Molar activity (MBq/nmol)	
		Low dose	High dose
[^125^I]I-Bapi-8D3_WT_	76 ± 5.7	30	0.89
[^125^I]I-8D3_D54A/Y92A_	74 ± 1.7	25	0.82
[^125^I]I-8D3_WT_	71	16	0.63

Values represent mean if n = 1 or mean ± standard deviation if n > 1

To increase the concentration for the high dose, cold antibody was supplemented to the radiolabelled antibody. The molar activity at each dose for the three antibodies are included in Table 2.

### Animals

All animal experiment procedures were approved by the Uppsala County Animal Ethics board (5.8.18–20,401/2020) and carried out according to the Swedish Animal Welfare Agency regulations and the European Communities Council Directive of 22 September 2010 (2010/63/EU). Animals were housed in an approved animal facility at Uppsala University with free access to food and water.

#### Pharmacokinetics and ex vivo biodistribution

Ex vivo biodistribution studies were performed on 6 to 7-month-old WT C57BL/6 mice (n = 3 per group; Fig. 1c). Mice under mild isoflurane sedation (Baxter Medical AB, Kista, Sweden) received a tail vein injection of [^125^I]I-Bapi-8D3_WT_, [^125^I]I-8D3_D54A/Y92A_ or [^125^I]I-8D3_WT_ with the doses in Table 3. Tail vein blood samples (8 µL) were taken 30 min and 2 h post-injection.

**Table 3:** Antibody dose (mg/kg) and radioactivity (MBq) administered to WT mice

Antibody	Low dose		High dose	
	Dose (mg/kg)	Radioactivity (MBq)	Dose (mg/kg)	Radioactivity (MBq)
[^125^I]I-Bapi-8D3_WT_	0.26 ± 0.023	1.2 ± 0.24	7.7 ± 0.10	1.4 ± 0.20
[^125^I]I-8D3_D54A/Y92A_	0.20 ± 0.025	0.95 ± 0.087	7.5 ± 0.32	1.3 ± 0.067
[^125^I]I-8D3_WT_	0.21 ± 0.023	0.87 ± 0.18	7.4 ± 0.74	0.91 ± 0.21

Values are mean ± standard deviation

Under isoflurane anaesthetics and immediately before euthanasia, a terminal cardiac blood sample was collected; half of which was centrifuged at 10,000 × g for 5 min and separated into plasma and blood cell pellet. Mice were euthanized 4 h post-injection via transcardial perfusion with 40 mL of 0.9% NaCl for 2.5 min. The left cortex was collected and capillary depletion was performed as previously described [16]. The right hemisphere, left midbrain and left cerebellum were isolated and frozen on dry ice. Lung, heart, liver, pancreas, spleen, kidney, femoral muscle, femoral bone, and skull were also isolated and a urine sample was collected. Radioactivity in all samples was measured with a 2480 Wizard™ γ-counter (PerkinElmer). Dose-normalized concentrations were calculated as the percent of injected dose of activity per gram tissue:

$$\%\text{ID}/\text{g}\hspace{0.17em}=\hspace{0.17em}\text{measured radioactivity per gram tissue }/\text{ total injected dose of radioactivity}\hspace{0.17em}\times \hspace{0.17em}100$$

### Statistical analyses

Statistical analyses were performed with GraphPad Prism 10.2.3. The EC50 values for the on-cell affinity assay were calculated from agonist concentration vs response curves with variable slope (four parameters). Results are reported as mean ± standard deviation unless otherwise mentioned. A one-way ANOVA with Tukey multiple comparisons of means was performed to determine significant differences in the brain-to-blood ratios, percent in blood fractions and percent in capillary depletion fractions, and the concentration of each antibody in brain regions and capillary depletion fraction of the three antibodies within each of the two dose groups. Differences between blood pharmacokinetic AUCs were assessed with Brown-Forsythe and the Welch ANOVA tests with Dunnett’s T3 multiple comparisons test.

## Results

### Two sets of monovalent and bivalent antibodies with matching apparent affinities were identified

All antibodies and fragments produced were successfully purified (Figure S1). In Biacore, it was not possible to determine affinity for six of the 12 Fab fragments screened (Figure S2). The 8D3_WT_ Fab and five of the variants that displayed weaker binding than the 8D3_WT_ Fab were selected for KiH production and further analysis. Three of these variants were selected from the six that had too weak binding in the hopes of finding variants with more than a twofold weaker affinity than the 8D3_WT_ Fab. The Langmuir 1:1 model did not fit many IgG curves well, including the 8D3_WT_ IgG, so apparent affinities for IgGs were estimated with a steady state affinity model instead (Figure S3, S4). Even with the steady state analysis, eight of the IgGs initially screened bound too weakly to determine an apparent affinity. Five IgG variants with apparent affinities similar to or weaker than the affinity of the 8D3_WT_ Fab were selected for KiH production and further analysis.

**Fig. 2:**
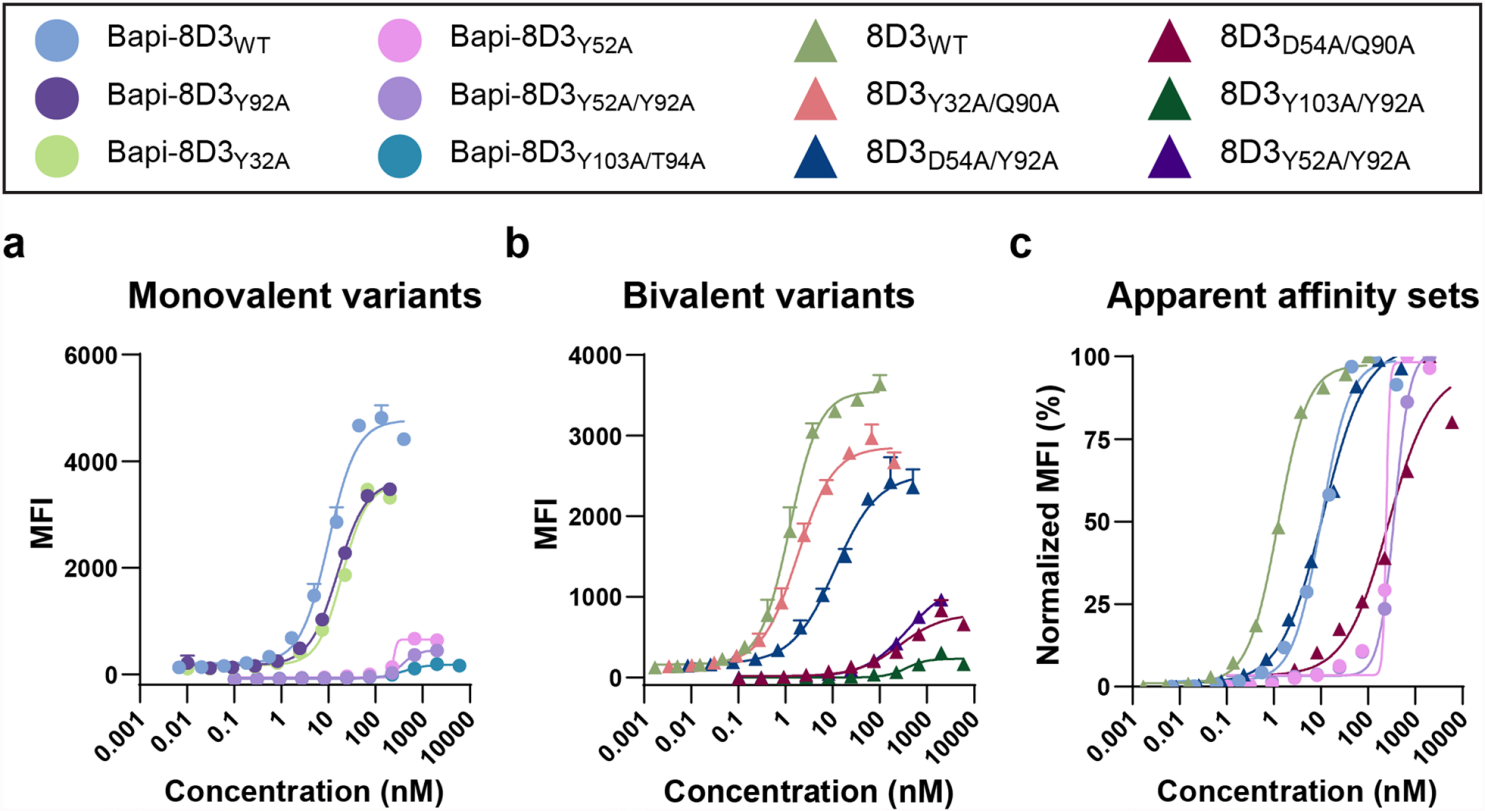
Apparent affinity determination on Sp2/0-Ag14 cells. Mean fluorescent intensity (MFI) from cell mTfR affinity assay with **a** monovalent and **b** bivalent KiH antibodies. **c** Overlap of the two apparent affinity sets and 8D3_WT_

Affinities of the monovalent antibodies separated into two groups when measured on Sp2/0-Ag14 cells: a higher apparent affinity group (Bapi-8D3_WT_, Bapi-8D3_Y92A_, and Bapi-8D3_Y32A_) at 10–20 nM and a lower apparent affinity group (Bapi-8D3_Y52A_, Bapi-8D3_Y52A/Y92A_, and Bapi-8D3_Y103A/T94A_) at 234–377 nM (Fig. 2a, Table 4). The on-cell affinities for the bivalent antibodies also separated into two groups: one at 1.2–11 nM (8D3_WT_, 8D3_Y32A/Q90A_, and 8D3_D54A/Y92A_) and another at 268–386 nM (8D3_D54A/Q90A_, 8D3_Y103A/Y92A_, and 8D3_Y52A/Y92A_; Fig. 2b, Table 4). From the cell assay, we were able to identify two sets with similar apparent affinities: 1) Bapi-8D3_WT_ with 8D3_D54A/Y92A_ at ~ 10 nM and 2) Bapi-8D3_Y52A/Y92A_ or Bapi-8D3_Y52A_ with 8D3_D54A/Q90A_ at ~ 250 nM (Fig. 2c).

**Table 4:** Affinity to mTfR on Sp2/0-Ag14 cells for monovalent and bivalent antibodies

Monovalent		Bivalent	
Antibody	EC50	Antibody	EC50
Bapi-8D3_WT_	10.0 *	8D3_WT_	1.23
Bapi-8D3_Y92A_	15.7	8D3_Y32A/Q90A_	1.80
Bapi-8D3_Y32A_	19.6	8D3_D54A/Y92A_	11.2 *
Bapi-8D3_Y52A/Y92A_	234 ^††^	8D3_D54A/Q90A_	268 ^††^
Bapi-8D3_Y52A_	237 ^††^	8D3_Y103A/Y92A_	368
Bapi-8D3_Y103A/T94A_	377	8D3_Y52A/Y92A_	386

Antibodies for each apparent affinity set are indicated with a symbol beside the EC50

*for the 10 nM apparent affinity

^††^for the 250 nM apparent affinity set

### The effects of valency and affinity on TfR-mediated transcytosis are dose-dependent

**Fig. 3:**
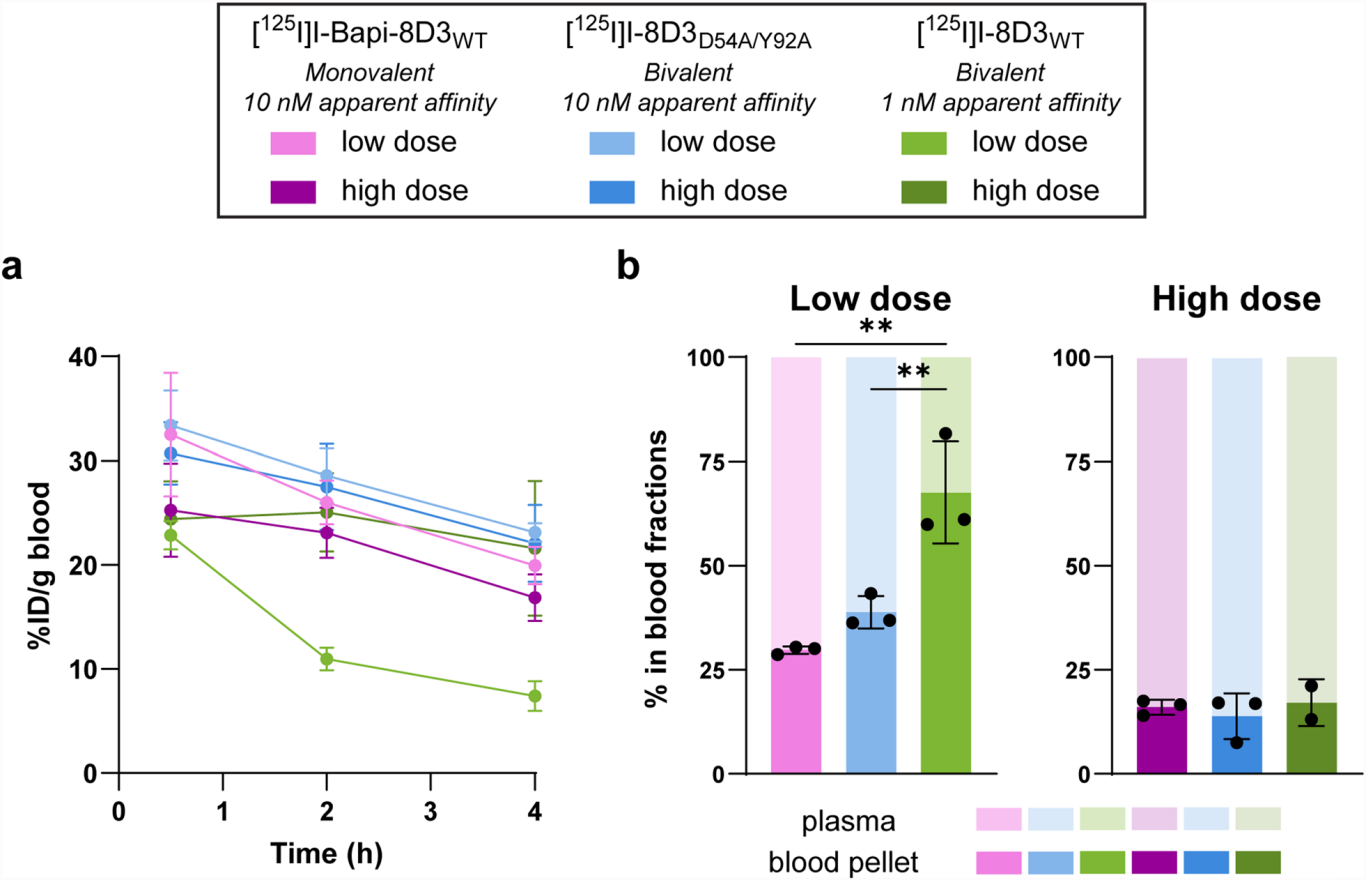
Ex vivo blood pharmacokinetics in WT mice 4 h post-injection. **a** Whole blood pharmacokinetics (%ID/g blood) of [^125^I]I-Bapi-8D3_WT_, [^125^I]I-8D3_D54A/Y92A_ and [^125^I]I-8D3_WT_ over 4 h post-injection at two doses. **b** Percent in blood fractions (blood pellet and plasma) 4 h post-injection. (** p ≤ 0.01)

The bivalent 8D3_WT_ (1 nM apparent affinity) and 10 nM apparent affinity set (monovalent Bapi-8D3_WT_ and bivalent 8D3_D54A/Y92A_) were radiolabelled with ^125^I to assess how valency and affinity affect biodistribution in vivo at a low dose of 0.22 ± 0.03 mg/kg and a high dose of 7.5 ± 0.43 mg/kg. The blood pharmacokinetic profiles over 4 h post-injection for each antibody at both doses were very similar except for [^125^I]I-8D3_WT_ which had a faster clearance (Fig. 3a, Table 5). The concentrations in plasma reflected the concentrations in the whole blood with the plasma concentration of [^125^I]I-Bapi-8D3_WT_ at the low dose being lower than either of the 10 nM apparent affinity antibodies at the low dose ([^125^I]I-Bapi-8D3_WT_, p ≤ 0.001; [^125^I]I-8D3_D54A/Y92A_, p ≤ 0.001) while all three antibodies had similar concentrations in the plasma at the high dose (Figure S5a). There were no significant differences in the concentrations in the blood pellet between antibodies within the two doses (Figure S5b).

**Table 5:** Area under the curve from the whole blood pharmacokinetics over 4 h post-injection (Fig. 3a)

Dose	[^125^I]I-Bapi-8D3_WT_	[^125^I]I-8D3_D54A/Y92A_	[^125^I]I-8D3_WT_
Low	89.8 (79.1–101)	98.1 (89.9–106)	43.7 (39.4–48.1) *
High	76.2 (66.3–86.1)	93.2 (80.0–106)	83.7 (67.1–100)

Values are written as mean (95% confidence interval). The * indicates that this value was significantly different (p ≤ 0.05) from all other values in the table

The relative distribution between plasma and blood pellet within the blood samples differed between doses (Fig. 3b). At the low dose, the percentage in the blood pellet for [^125^I]I-8D3_WT_ was higher than that for [^125^I]I-Bapi-8D3_WT_ (p = 0.002) and [^125^I]I-8D3_D54A/Y92A_ (p = 0.008). The percentage of antibody in the blood pellet decreased with the high dose for all three antibodies, but with no significant differences between the antibodies.

**Fig. 4:**
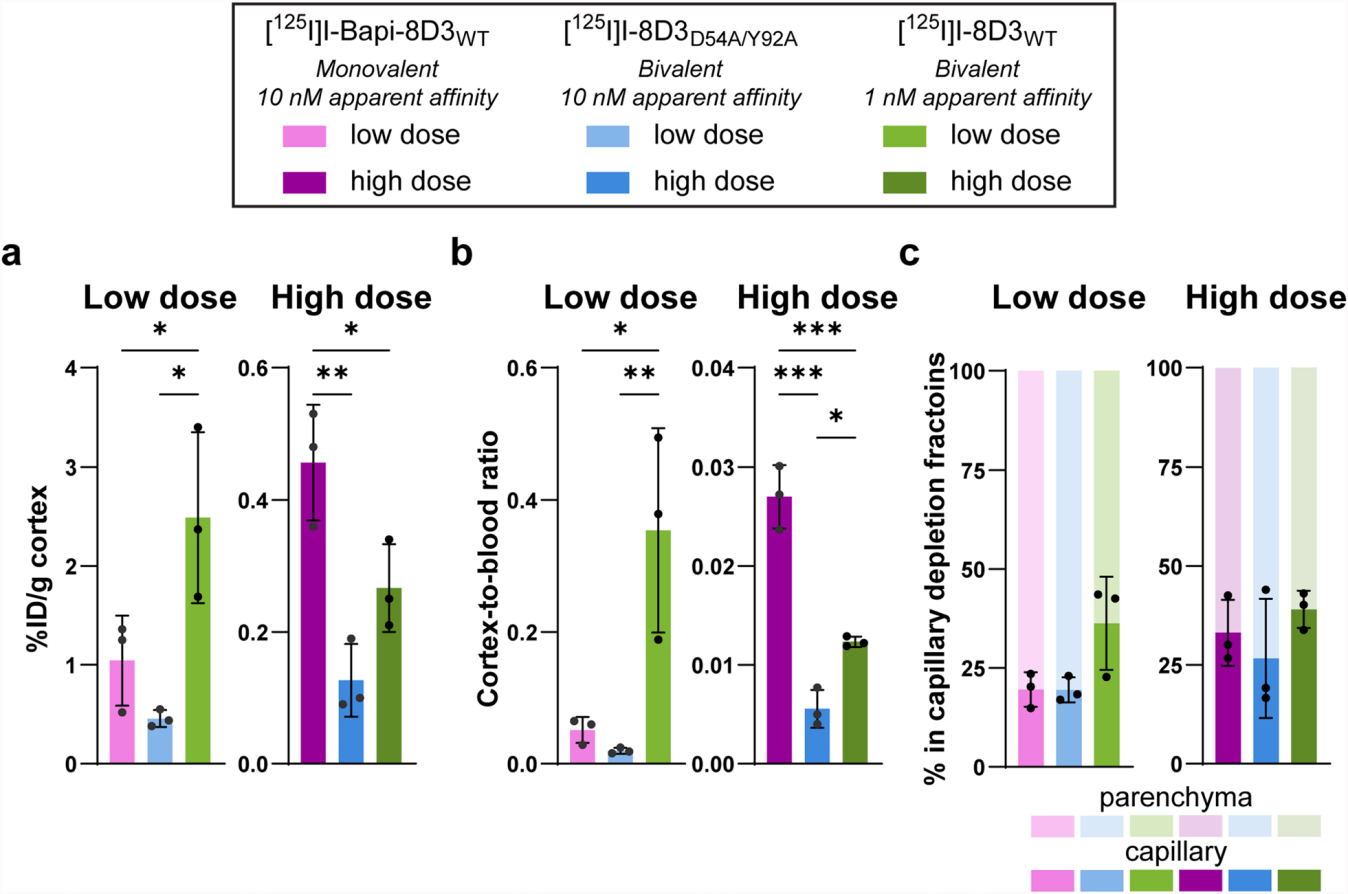
Antibody concentrations in cortex of WT mice 4 h post-injection. **a** Concentration in the cortex (%ID/g) and **b** cortex-to-blood ratio 4 h post-administration. **c** Percent in capillary depletion fractions (capillaries and parenchyma) 4 h post-administration. (* p ≤ 0.05, ** p ≤ 0.01, *** p ≤ 0.001

Each antibody had higher dose-normalized concentrations in the cortex when administered at the low dose compared to the high dose (Fig. 4a). At the low dose, the cortical concentration of [^125^I]I-8D3_WT_ was higher than that of both [^125^I]I-Bapi-8D3_WT_ (2.4-fold, p = 0.046) and [^125^I]I-8D3_D54A/Y92A_ (5.4-fold, p ≤ 0.011). While at the high dose, the monovalent TfR binder, [^125^I]I-Bapi-8D3_WT_, had higher cortical uptake than both bivalent 8D3 antibodies (3.6-fold for [^125^I]I-8D3_D54A/Y92A_, p = 0.003 and 1.7-fold with [^125^I]I-8D3_WT_, p = 0.039). These differences in antibody concentrations in the cortex were mirrored in the cortex-to-blood ratios (Fig. 4b). The concentrations in the cortex and cortex-to-blood ratios of the three antibodies at both doses were also representative of the other brain regions, as determined by measuring the full right hemisphere (Figure S6a-b).

Capillary depletion was performed to further investigate the distribution of antibody within the cortex. The antibody concentrations in the capillaries and parenchyma also followed the same trends as the concentrations of the whole cortex (Figure S7a-b). Although no significant differences were measured between the relative distribution of antibodies in the capillaries and parenchyma, some trends can be noted (Fig. 4c, Table 6). Firstly, the percentage in the capillaries of [^125^I]I-8D3_WT_ appeared to be higher than that of [^125^I]I-Bapi-8D3_WT_ and [^125^I]I-8D3_D54A/Y92A_ (1.9-fold for both) at the low dose. This distribution trend between antibodies at the low dose did not appear at the high dose. At the high dose, all three antibodies had a similar percentage in the capillaries. Finally, the percentage in the capillaries for all three antibodies at the high dose was similar to that of [^125^I]I-8D3_WT_ at the low dose.

**Table 6:** Distribution (%) of the antibodies in the capillary fractions from capillary depletion

Dose	[^125^I]I-Bapi-8D3_WT_	[^125^I]I-8D3_D54A/Y92A_	[^125^I]I-8D3_WT_
Low	19.6 ± 4.3	19.4 ± 3.2	36.3 ± 12
High	33.1 ± 8.4	26.6 ± 15	39.0 ± 4.7

Values are mean ± standard deviation

**Fig. 5:**
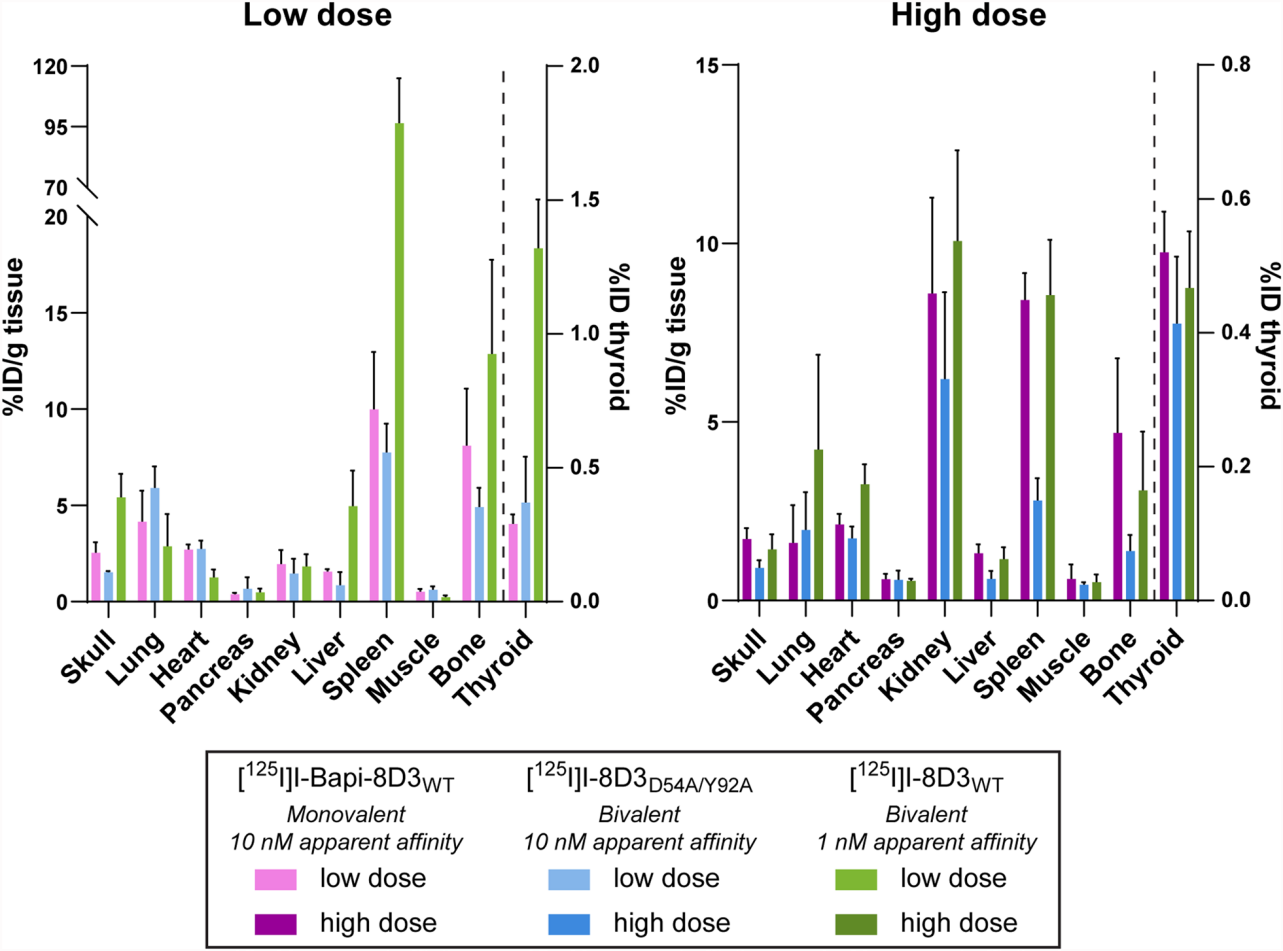
Peripheral biodistribution in WT mice 4 h post-injection. Concentrations in the thyroid (%ID) and other peripheral tissues (%ID/g) at a low or high dose

Finally, the peripheral biodistribution was assessed (Fig. 5). At the low dose, the concentrations of the 10 nM apparent affinity antibodies, [^125^I]I-Bapi-8D3_WT_ and [^125^I]I-8D3_D54A/Y92A_, were similar in all peripheral samples collected. Although not statistically verified, the concentration of the 10 nM apparent affinity antibodies were also lower than the concentration of [^125^I]I-8D3_WT_ in the skull, liver, spleen, bone and thyroid at the low dose. At the high dose, the three antibodies distributed similarly to each other in all samples collected, except for in the liver, spleen and bone where the concentration of [^125^I]I-8D3_D54A/Y92A_ was lower than the other two antibodies (although also not verified with statistical analysis).

## Discussion

Previous studies have investigated the effects of valency by comparing the same antibody in a monovalent and bivalent format [3, 9, 16, 19, 20, 22]. However, this typically leads to a difference in apparent affinity as well which can influence TfR-mediated transcytosis efficiency. To isolate the effects of valency on TfR-mediated transcytosis, multiple 8D3 affinity variants were produced in both monovalent and bivalent formats to find full-sized, monovalent and bivalent antibodies with similar apparent affinity. Two apparent affinity sets were determined from the original 12 Fab fragments and 29 IgGs assessed. The 10 nM apparent affinity set and the WT bivalent antibody were tested in vivo at two different doses. Brain uptake via TfR-mediated transcytosis is affected by dose, apparent affinity and valency. Furthermore, the effects of apparent affinity and valency are dose-dependent with apparent affinity having more influence at lower doses and valency becoming more important as the dose increases.

In order to produce the affinity variants used here, previously described alanine point mutations were introduced in the CDRs of 8D3 [12, 15]. This is the first time to our knowledge that these mutations have been produced and characterized as monovalent binders. To screen the affinity of the variants *in* vitro, an immobilized mTfR setup in Biacore was used as this setup allows for a comparison of apparent affinities between monovalent and bivalent binders [27]. However, the 1:1 model did not fit the bivalent data to an acceptable degree. mTfR immobilized on the chip surface has proven to be sensitive to immobilization and repeated regeneration cycles [27]. Therefore, we used a steady state affinity analysis on the bivalent variants to determine an approximate apparent affinity for our first round of variant selection. The ranking of relative apparent affinity from the Biacore screening data correlated well with the Sp2/0-Ag14 cell data.

Monovalent and bivalent forms of 8D3 affinity variants were produced as full-length, antibodies using KiH technology for the further characterization on Sp2/0-Ag14 cells and in vivo pharmacokinetic study. The KiH design employed here kept the size and Fc domain constant between monovalent and bivalent binders as both size and Fc domain can impact blood pharmacokinetics, brain uptake, peripheral tissue biodistribution, and rates of elimination [16, 29]. While exploring the impact of size on brain delivery using Fab fragments could provide additional insights from the initial investigations of Faresjö and colleagues, it poses certain challenges. Fab fragments lack the Fc domain, which interacts with the neonatal Fc receptor (FcRn) and influences brain uptake [29]. Addressing this would require redesigning the bivalent antibodies with specific mutations to reduce FcRn binding, such as H310A and H435Q [29, 30]. Thus, while further investigation into size effects remains valuable, the KiH design with alanine point mutations allowed isolation of the effects of valency and apparent affinity without other confounding variables.

Throughout the study, the dose-normalized biodistribution of all three antibodies differed between doses. Each antibody had higher dose-normalized concentrations in the brain and peripheral tissues with high levels of TfR (spleen, liver, and bone) when administered at the low dose compared to the high dose. This trend of lower dose-normalized tissue uptake with a higher dose has been previously reported and is likely due to TfR saturation [3, 9, 31]. Although a higher absolute amount of antibody enters the brain at higher doses compared to lower doses, the efficiency of this delivery decreases at higher doses due to TfR-saturation. As a result, a smaller proportion of administered antibody enters the brain at higher doses. Comprehensive dose–response data to determine optimal dosing for the variants was not provided since the primary aim of this study was to evaluate how various factors influence brain uptake and the measured response in a dose–response curve would vary depending on the TfR-binding construct’s intended use (i.e. for therapeutic or diagnostic purposes). Moreover, brain concentrations and biodistribution were measured at a single time point (4 h post-injection) and thus, this study only provides a momentary view of brain delivery. This early time point was chosen based on historical data that indicates that TfR-mediated brain concentrations peak around 4 h post-injection in mice without any intrabrain target for the antibody to bind to [3, 9, 16]. Further evaluation of brain concentrations over multiple time points would provide a more comprehensive understanding of the pharmacokinetics and biodistribution profiles; leading to insights for optimal windows for imaging with low doses and therapeutic efficacy with high doses and thus, improving diagnostic and therapeutic applicability.

It has also previously been reported that for a bispecific antibody, the concentration in total brain tissue is not necessarily an exact representation of the antibody available in the parenchyma to bind to the intrabrain target [16]. The concentration in the brain tissue is a sum of the concentration of bispecific antibody still bound to TfR in the capillaries and the concentration of bispecific antibody in the parenchyma. Thus, capillary depletion of the cortex was performed to further investigate the concentration of target-available antibody in the parenchyma. This technique further supported the idea that TfR saturation is more likely to occur at a higher dose with the trend that both 10 nM apparent affinity antibodies ([^125^I]I-Bapi-8D3_WT_ and [^125^I]I-8D3_D54A/Y92A_) had higher capillary retention at the high dose than at the low dose.

Analysis within each dose suggests that different factors influence biodistribution of TfR-binding antibodies depending on the dose. While a larger sample size may enhance statistical power and address inter-individual variability, we could observe the following. At the low dose, apparent affinity seemed to have more of an effect in determining biodistribution than valency. [^125^I]I-8D3_WT_ had much quicker elimination from blood and lower fraction in plasma 4 h post-injection than either of the 10 nM apparent affinity antibodies ([^125^I]I-Bapi-8D3_WT_ and [^125^I]I-8D3_D54A/Y92A_). This was likely due to the higher apparent affinity binding to TfR at the BBB and on other peripheral tissue quicker, thus clearing the antibodies from blood quicker [31, 32]. Effects in the blood due to TfR-mediated clearance from the blood have also been shown to be more apparent at lower doses as the TfR on tissue surfaces is saturable [33]. The concentration of [^125^I]I-8D3_WT_ at the low dose was also higher in the brain, peripheral tissues with high levels of TfR (spleen, liver, and bone), and in both the capillary and parenchymal fractions after capillary depletion. However, when looking at the proportional distribution between the two capillary depletion fractions, there was a trend that [^125^I]I-8D3_WT_ was trapped in the capillaries to a higher extent. Although not significant, the capillary depletion values accurately replicated the previously reported distribution of bispecific antibodies with monovalent and bivalent 8D3_WT_ binders [16].

At the high dose, valency seemed to have a larger influence on the brain uptake of a TfR-binding antibody. The monovalent [^125^I]I-Bapi-8D3_WT_ had higher brain uptake and concentrations in the parenchyma. This difference between the monovalent antibody and the two bivalent antibodies was expected due to the commonly accepted idea that at a high, therapeutic dose, monovalent binding to TfR is a key criterion for efficient transport across the BBB [3, 19]. The 1:2 binding stoichiometry of bivalent binders is considered to make them prone to binding two different TfR, leading to TfR clustering on the endothelial cell surface and driving the endosomes to lysosomal degradation [19–22, 34]. It was, however, unexpected that the higher brain uptake with the monovalent antibody did not translate to a lower proportion of antibody being trapped in the capillaries in capillary depletion compared to the bivalent antibodies. It has also been suggested that there is an optimal affinity window for maximized BBB transport at therapeutic doses [9, 14] and it may be that with the TfR saturation that occurred from the high dose, the apparent affinities of the antibodies studied here were too similar to be able to detect subtle differences with the capillary depletion technique. The other apparent affinity set (Bapi-8D3_Y52A/Y92A_ or Bapi-8D3_Y52A_ with 8D3_D54A/Q90A_) was not tested in vivo in this study as the apparent affinity of ~ 250 nM would result in too low brain concentrations to determine meaningful differences in the ex vivo analysis at the low dose. The affinity set could serve as a meaningful surrogate for examining the mechanistic effects of valency and affinity at the high dose, as the mTfR on-cell affinity is within a two-fold range of the human TfR Biacore affinity observed in variants currently under clinical evaluation [35]. However, this set with an apparent affinity of 250 nM would necessitate a multi-timepoint analysis, which introduces additional complexity. Nonetheless, a focused, in-depth mechanistic analysis of this apparent affinity set with multiple timepoints and multiple high doses could provide valuable insights in future explorations.

## Conclusions

In conclusion, we successfully produced and screened a panel of monovalent and bivalent affinity variants of the anti-mTfR antibody, 8D3. From this panel, two sets of monovalent and bivalent 8D3 formats that bind to mTfR on Sp2/0-Ag14 cells with similar apparent affinities were identified. Upon intravenous injection, the effect of dose on brain uptake was confirmed such that there is lower brain uptake with a higher dose. The effects of valency and apparent affinity were also dose-dependent. At a low dose, apparent affinity had greater influence on brain uptake than valency as the higher apparent affinity bivalent binder demonstrated higher brain uptake compared to lower affinity binders independent of valency. The effect of valency was more important at the high dose; such that the monovalent antibody had higher brain uptake than the two bivalent antibodies at the high dose. Therefore, the purpose of a bispecific, TfR-binding antibody will influence its design; a diagnostic antibody which will be dosed lower will likely benefit from a higher apparent affinity, while a therapeutic antibody being dosed higher will benefit from monovalent TfR-binding.

## Supplementary Information

Supplementary material 1.

## Data Availability

The datasets generated and/or analysed during the current study are available from the corresponding author on reasonable request.

## Abbreviations

^125^I: Iodine-125
Bapi: Bapineuzumab
BBB: Blood–brain barrier
CNS: Central nervous system
KiH: Knob-into-hole
MFI: Mean fluorescent intensity
mTfR: Mouse transferrin receptor 1
PBS: Phosphate buffered saline
TfR: Transferrin receptor
WT: Wild-type
