# Supplementary material for: The effects of dose, valency, and affinity on TfR-mediated brain delivery in vivo
Source: Fluids Barriers CNS. 2025 Apr 8;22:36. doi: 10.1186/s12987-025-00643-y (PMC11980351; doi:10.1186/s12987-025-00643-y)
Supplement: Supplementary file 1 — Supplementary material 1. [file 12987_2025_643_MOESM1_ESM.docx]

**Table S1.** 8D3 Fab fragment point mutations with CDR location for initial screen.

| **8D3 Fab variants** | **Heavy chain mutations** | | | **Light chain mutations** | | | **References** |
| --- | --- | --- | --- | --- | --- | --- | --- |
|  | **CDR1** | **CDR2** | **CDR3** | **CDR1** | **CDR2** | **CDR3** |  |
| 8D3_WT_ Fab | -- | -- | -- | -- | -- | -- | Boado ’09 |
| 8D3 Fab 1 | -- | -- | -- | -- | -- | Y92A | Do 2020 |
| 8D3 Fab 2 | -- | -- | S101A | -- | -- | -- | Do 2020 |
| 8D3 Fab 3 | -- | Y52A | -- | -- | -- | -- | Do 2020 |
| 8D3 Fab 4 | -- | Y52A | -- | -- | -- | Y92A | Do 2020 |
| 8D3 Fab 5 | -- | Y52A | S101A | -- | Y49A | Y92A | Do 2020 |
| 8D3 Fab 6 | -- | -- | Y103A | -- | -- | T94A | Webster |
| 8D3 Fab 7 | -- | -- | Y103A | -- | -- | Q90A | Webster |
| 8D3 Fab 8 | -- | -- | Y103A | -- | -- | W96A | Webster |
| 8D3 Fab 9 | Y32A | -- | -- | -- | -- | -- | Do 2020 |
| 8D3 Fab 10 | -- | D54A | -- | -- | -- | -- | Do 2020 |
| 8D3 Fab 11 | -- | -- | -- | D28A | -- | -- | Do 2020 |

**Table S2.** Heavy and light chain mutation pairings for 8D3 IgGs in initial screen.

| **8D3 IgG variants** | **Heavy chain** | **Light chain** | **References** |
| --- | --- | --- | --- |
| 8D3_WT_ IgG | WT | WT | Boado ’09 |
| 8D3 IgG 1 | WT | D28A | Do 2020 |
| 8D3 IgG 2 | WT | Q90A | Webster 2017 |
| 8D3 IgG 3 | WT | T94A | Webster 2017 |
| 8D3 IgG 4 | WT | W96A | Do 2020 |
| 8D3 IgG 5 | WT | Y92A | Webster 2017 |
| 8D3 IgG 6 | D54A | WT | Do 2020 |
| 8D3 IgG 7 | D54A | D28A | Do 2020 |
| 8D3 IgG 8 | D54A | Q90A | Do + Webster |
| 8D3 IgG 9 | D54A | T94A | Do + Webster |
| 8D3 IgG 10 | D54A | W96A | Do 2020 |
| 8D3 IgG 11 | D54A | Y92A | Do + Webster |
| 8D3 IgG 12 | Y32A | WT | Do 2020 |
| 8D3 IgG 13 | Y32A | D28A | Do 2020 |
| 8D3 IgG 14 | Y32A | Q90A | Do + Webster |
| 8D3 IgG 15 | Y32A | T94A | Do + Webster |
| 8D3 IgG 16 | Y32A | W96A | Do 2020 |
| 8D3 IgG 17 | Y32A | Y92A | Do + Webster |
| 8D3 IgG 18 | Y52A | WT | Do 2020 |
| 8D3 IgG 19 | Y52A | D28A | Do 2020 |
| 8D3 IgG 20 | Y52A | Q90A | Do + Webster |
| 8D3 IgG 21 | Y52A | W96A | Do 2020 |
| 8D3 IgG 22 | Y52A | Y92A | Do + Webster |
| 8D3 IgG 23 | Y103A | WT | Do + Webster |
| 8D3 IgG 24 | Y103A | D28A | Do + Webster |
| 8D3 IgG 25 | Y103A | Q90A | Webster |
| 8D3 IgG 26 | Y103A | T94A | Webster |
| 8D3 IgG 27 | Y103A | W96A | Do + Webster |
| 8D3 IgG 28 | Y103A | Y92A | Webster |

**
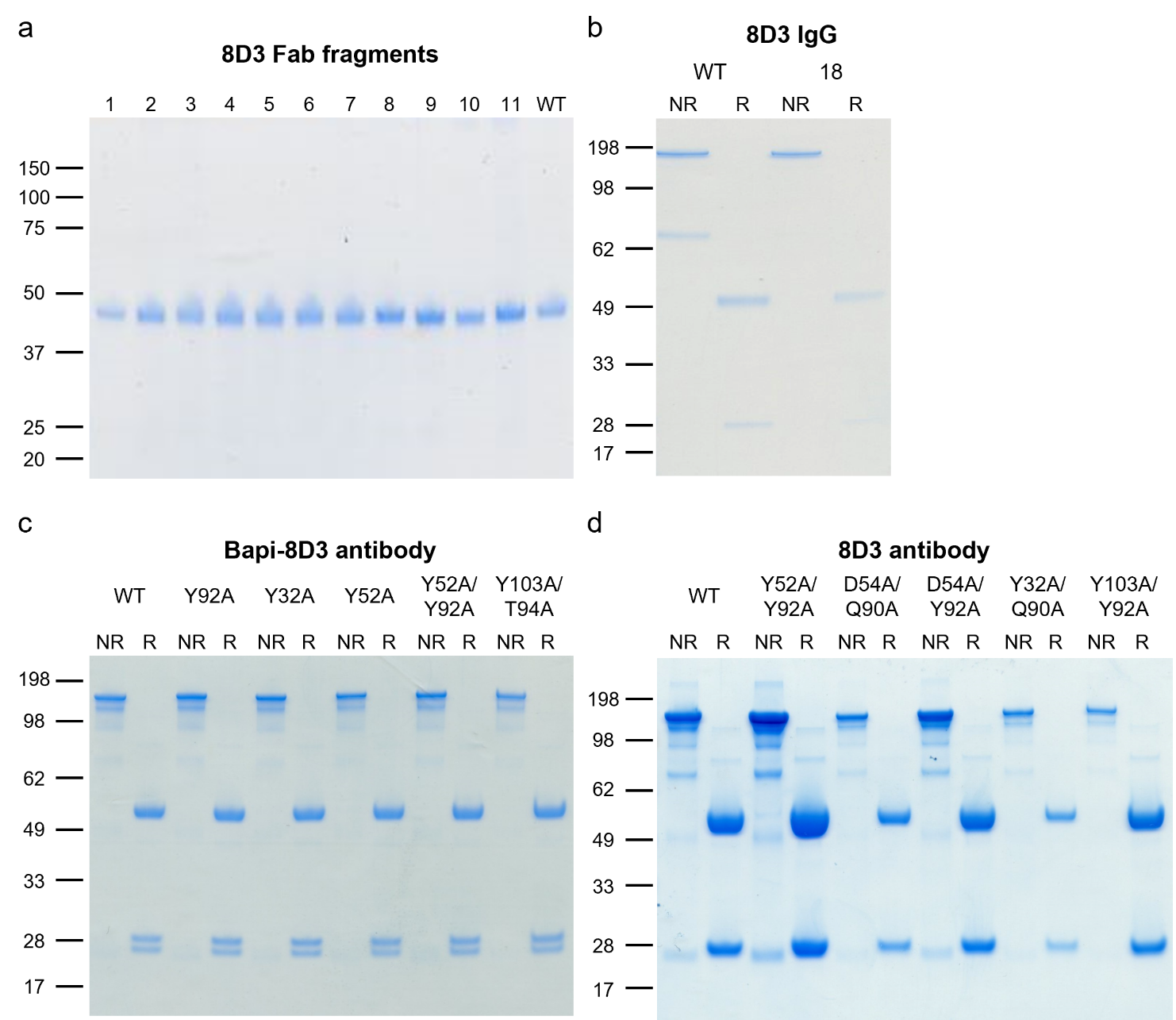
**

**Figure S1.** SDS-PAGE analysis of the 12 Fab fragments (**a**), a selected two of the 29 scanned IgGs (**b**), the six monovalent Bapi-8D3 KiH IgGs (**c**) and the six bivalent 8D3 KiH IgGs (**d**). The full-length IgGs were analyzed in both non-reducing (NR) and reducing (R) conditions.

**
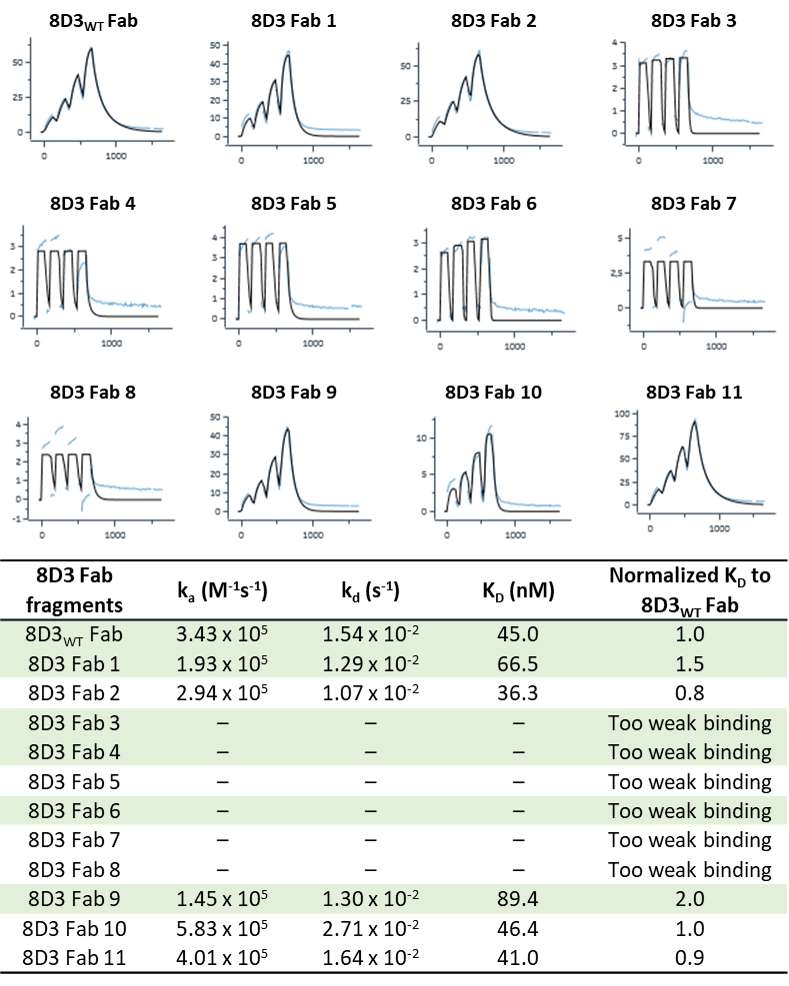
**

**Figure S2.** Affinity analysis of 8D3 alanine point mutation Fab fragments in Biacore. Representative sensorgrams with blank subtracted data in blue and 1:1 fitted curves in black. The table indicates the kinetic rate constants (k_a_ and k_d_), equilibrium dissociation constant (K_D_) and affinity relative to 8D3 Fab WT for each Fab fragment. Highlighted in green are the Fab fragments selected for further analysis.


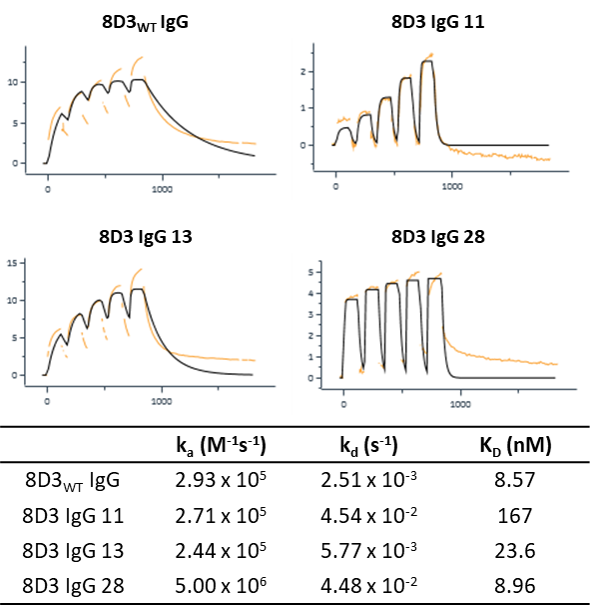


**Figure S3.** Affinity analysis of selected 8D3 IgG bivalent variants in Biacore. Representative sensorgrams with blank subtracted data in yellow with 1:1 fitted curve in black. The table indicates the kinetic rate constants (k_a_ and k_d_) and equilibrium dissociation constant (K_D_).


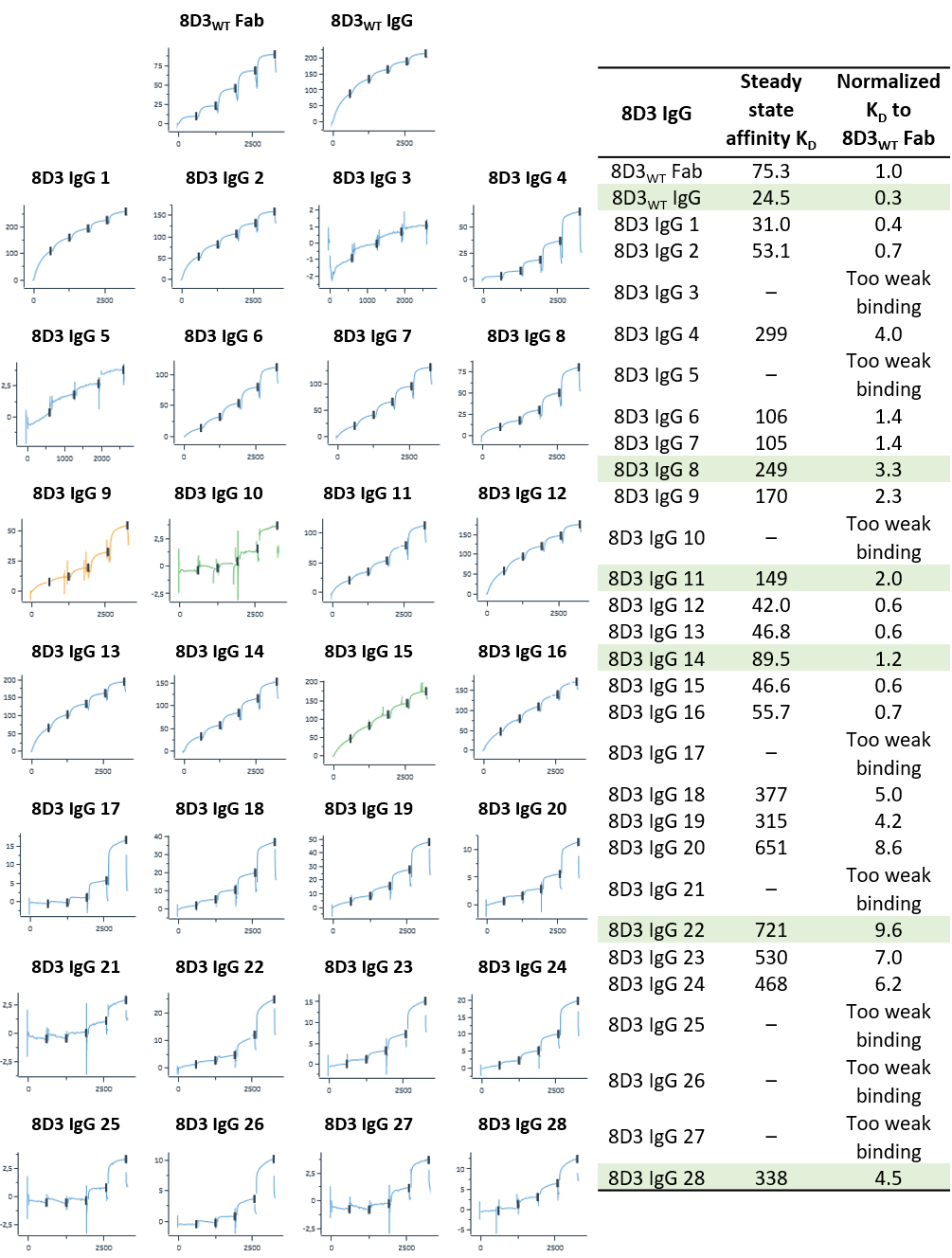


**Figure S4.** Affinity analysis of 8D3 alanine point mutation IgGs in Biacore. Representative sensorgrams with blank subtracted data with steady state affinity positions represented by the black ticks. Although steady state was not reached, data was analyzed with a steady state affinity method since this evaluated the affinity better than a 1:1 curve fit. The table indicates the steady state affinity K_D_ and affinity relative to 8D3 Fab WT for each IgG with the IgGs selected for further analysis highlighted in green.

**
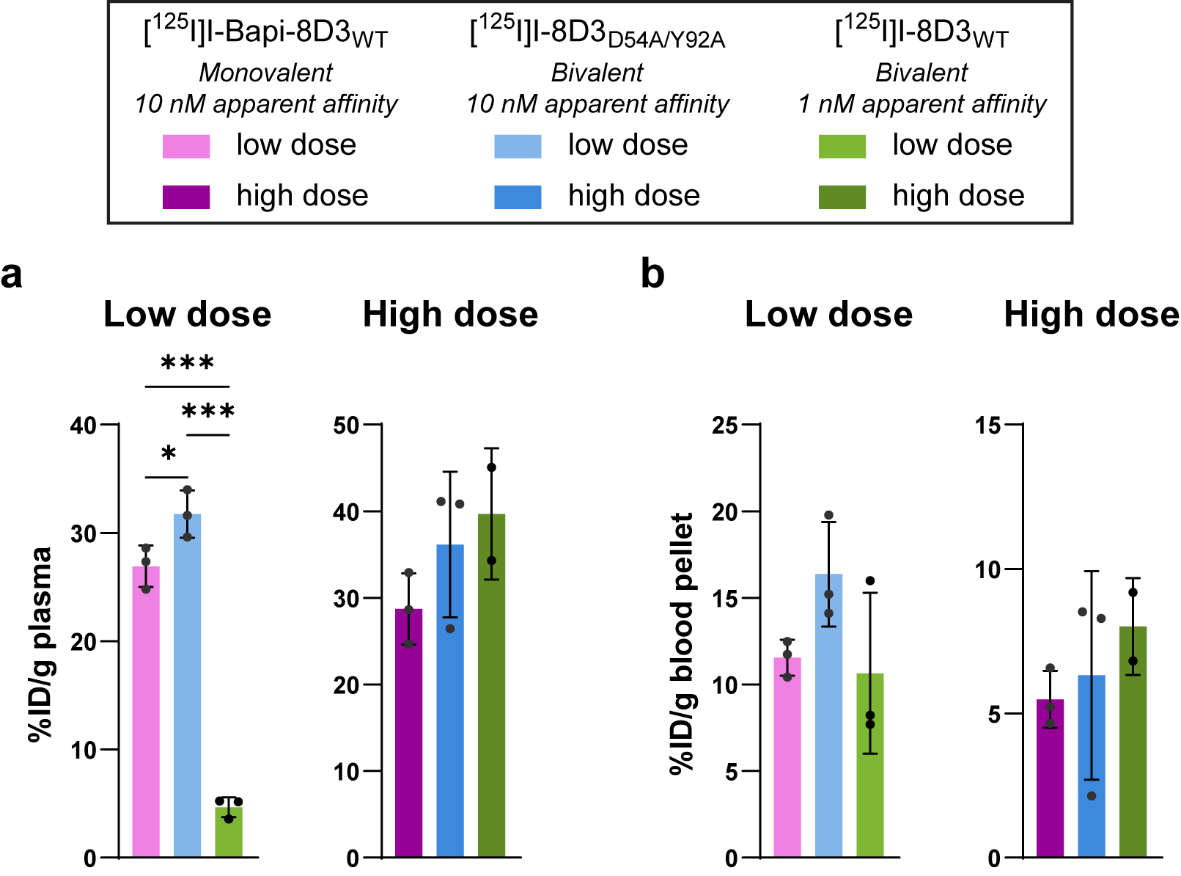
**

**Figure S5.** Concentrations of injected antibody in the (**a**) plasma (%ID/g plasma) and (**b**) blood pellet (%ID/g blood pellet). (* p ≤ 0.05, ** p ≤ 0.01, *** p ≤ 0.001).


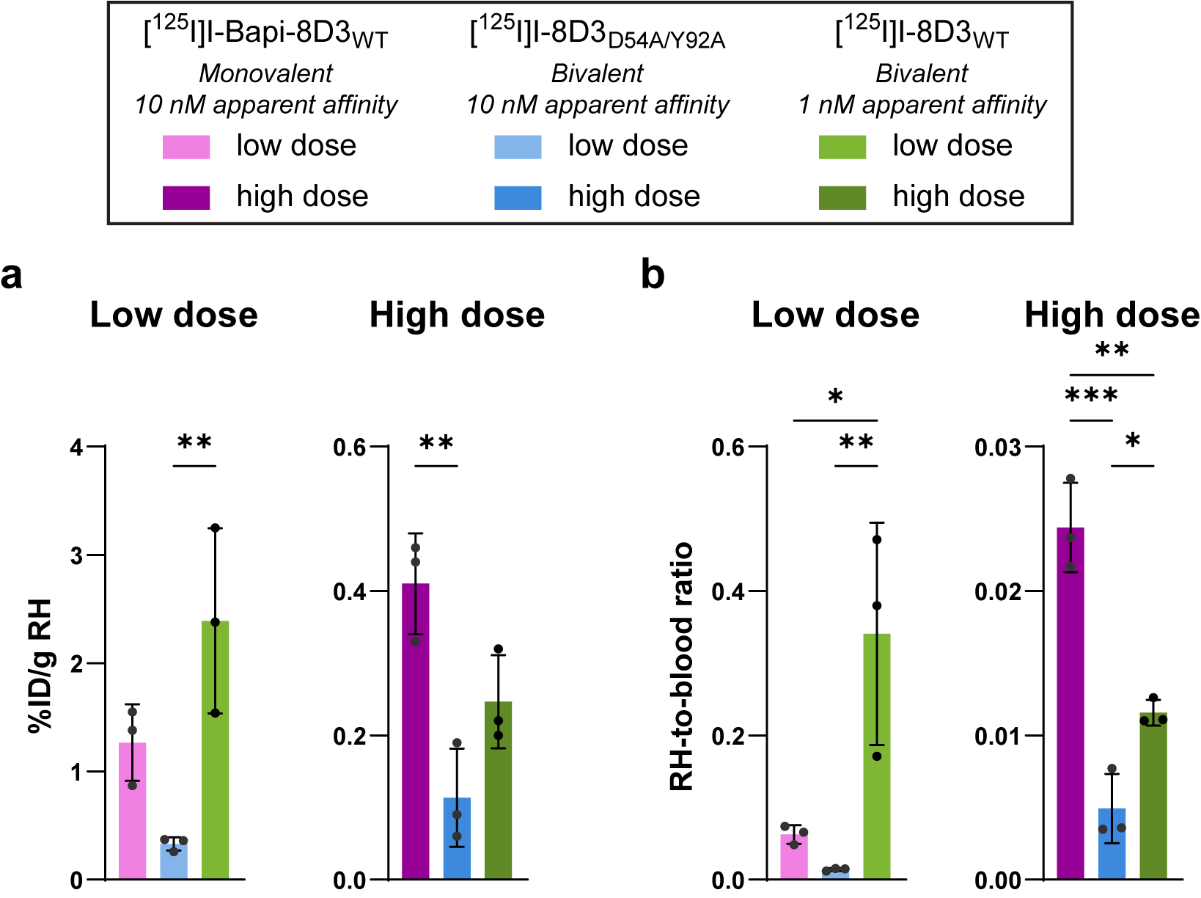


**Figure S6.** (**a**) Concentration (%ID/g) in the right hemisphere (RH) and (**b**) right hemisphere-to-blood ratio 4 h post-administration. (* p ≤ 0.05, ** p ≤ 0.01, *** p ≤ 0.001).


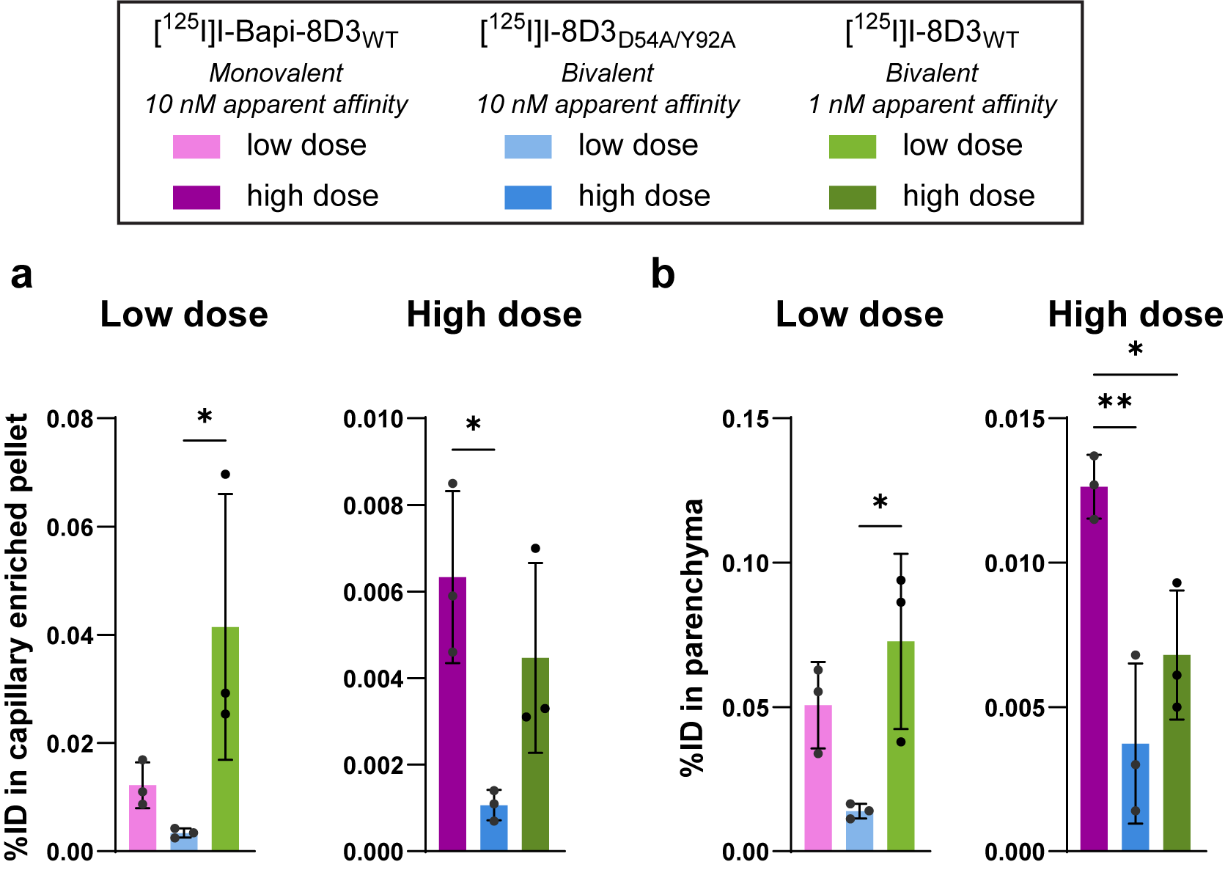


**Figure S7.** Concentrations (%ID) in the **(a)** capillary enriched pellet and **(b)** parenchyma from capillary depletion of cortices at 4 h post-administration. (* p ≤ 0.05, ** p ≤ 0.01).
